# Distribution of adenosine receptors in human sclera fibroblasts

**Published:** 2008-03-14

**Authors:** Dongmei Cui, Klaus Trier, Xiang Chen, Junwen Zeng, Xiao Yang, Jianmin Hu, Jian Ge

**Affiliations:** 1 State Key Laboratory of Ophthalmology, Zhongshan Ophthalmic Center, Sun Yat-sen University, Guangzhou, China; 2 Trier Research Laboratories, Tingskiftevej 6, DK-2900 Hellerup, Denmark

## Abstract

**Purpose:**

Systemic treatment with adenosine receptor antagonists has been reported to affect the biochemistry and ultrastructure of rabbit sclera. This study was conducted to determine whether adenosine receptors (ADORs) are present in human scleral fibroblasts (HSF).

**Methods:**

Primary HSF were cultured in vitro and identified with anti-vimentin, anti-keratin, anti-desmin, and anti-S-100 antibodies. Confocal fluorescence microscopy was used to study the distribution of ADORs in the HSF cell lines and in the frozen human scleral sections. ADOR protein expression in HSF and human sclera was confirmed by western blot analysis of cell lysates.

**Results:**

ADORs were expressed in both HSF and human sclera. This was confirmed by western blot. ADORA1 expression was concentrated in the nucleus. ADORA2A was concentrated mainly in one side of the cytoplasm, and ADORA2B was found both in the nucleus and the cytoplasm. ADORA3 was expressed weakly in the cytoplasm.

**Conclusions:**

All four subtypes of ADOR were found in HSF and may play a role in scleral remodeling.

## Introduction

Scleral fibroblasts are involved in scleral remodeling during axial elongation in myopia. In myopic eyes, the sclera is thinner and the diameter of collagen fibrils in the posterior sclera are smaller compared with normal eyes [1-4]. Chronic treatment of rabbits with the non-selective adenosine receptor (ADOR) antagonist, 7-methylxanthine, results in a significant increase in the scleral concentration of collagen and an increase in the diameter of the collagen fibrils in the posterior sclera [5]. Furthermore, results from a small scale clinical trial suggest that systemic treatment with 7-methylxanthine slows down the elongation rate of the eye in myopic children [6]. It is therefore possible that ADORs play a role in scleral remodeling in progressive myopia.

ADORs belong to the superfamily of guanine nucleotide-binding G-protein-coupled receptors that are ubiquitously expressed in a wide variety of tissues. The family includes four receptor subtypes, ADORA1, ADORA2A, ADORA2B, and ADORA3 [7].

ADORA1 and ADORA2 have been associated with the regulation of the intraocular pressure in rabbits [8] and monkeys [9]. ADORA1, ADORA2A, and ADORA2B mRNA have been demonstrated to be present in the ciliary processes and retina of rat eyes, although no significant expression of ADORA3 mRNA was found [10]. Until now, the presence of ADORs in the human sclera has not been confirmed. This study was conducted to determine whether ADORs are present in human scleral fibroblasts (HSF).

**Figure 1:**
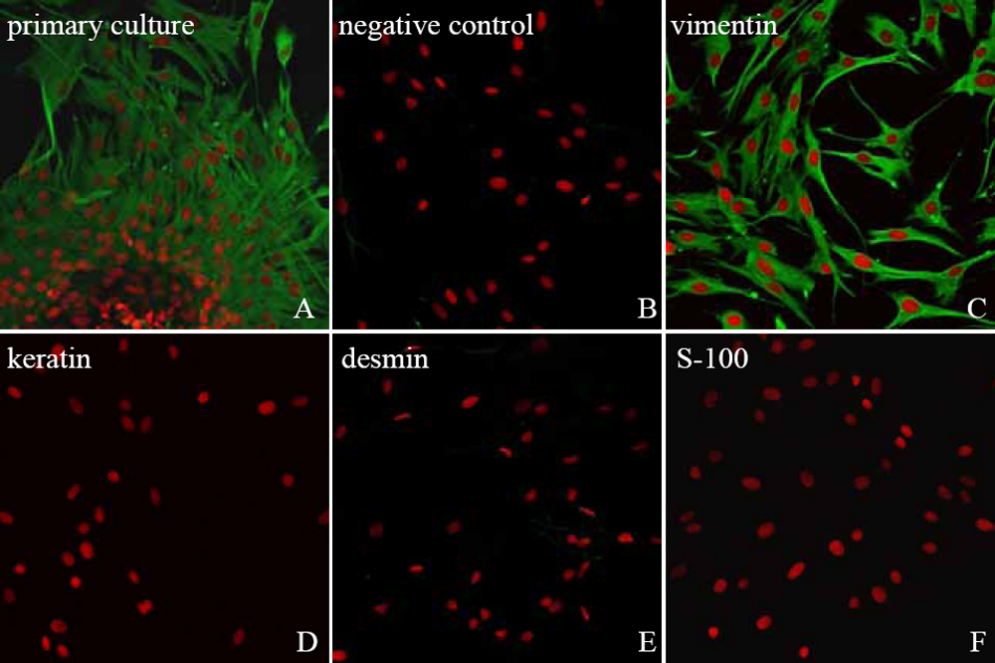
Identification of HSF with vimentin, keratin, desmin, and S-100. PI dyed the nucleus (red) and the FITC-marked secondary antibody (green). **A**: Vimentin antibody was added to primary cultured HSF migrating from a piece of sclera tissue. **B**: Negative controls used FITC-marked secondary antibodies of HSF. **C**: Vimentin is strongly expressed in the cytoplasm of HSF. **D**,**E**,**F**: Keratin, desmin, and S-100 protein are not expressed in the cytoplasm or nucleus of HSF. Magnification: 200X.

## Methods

### Materials

Dulbecco’s modified Eagle's medium plus Ham’s nutrient mixture F-12 (DMEM/F12), Hank's balanced salt solution (HBSS), fetal bovine serum (FBS), phosphate buffered saline (PBS), and trypsin were purchased from Gibco (Grand Island, NY). Plastic tissue culture dishes, chamber slides, and 24 well plates were obtained from Corning (Corning Ltd, Tokyo, Japan). 1X antibiotic/antimycotic (penicillin-streptomycin solution) was obtained from Invitrogen (Invitrogen Corp, Carlsbad, CA). Anti-ADORA1, anti-ADORA2A, anti-ADORA2B, and anti-ADORA3 antibodies (rabbit pAb) were obtained from Chemicon (Chemicon International Inc., Temecula, CA). Anti-vimentin, anti-keratin, anti-desmin, anti-S-100 antibodies, and fluorescent secondary antibody were obtained from Zhongshan Goldenbridge (Zhongshan Goldenbridge Biotechnology Co Ltd, Bejing, China). BCA kits were obtained from Shenneng Bocai (Shenneng Bocai Biotechnology Co. Ltd, Shanghai, China). Propidium iodide (PI), optimum cutting temperature compound (OCT), and other chemicals were purchased from Sigma (St. Louis, MO).

**Figure 2:**
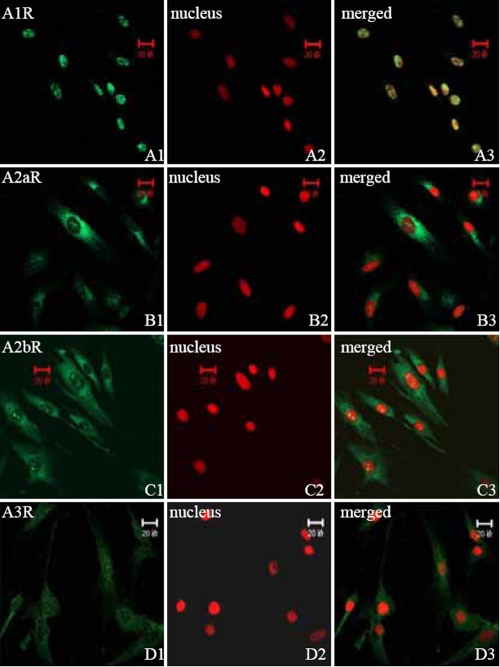
Distribution of ADORA1, ADORA2A, ADORA2B, and ADORA3 in HSF in vitro using indirect immunofluorescence. FITC-marked the secondary antibody (green; 1) and PI dyed the nucleus (red; 2). (1) and (2) are combined into (3). ADORA1 is concentrated in the nucleus of HSF (A1-A3). ADORA2A is mainly distributed on one side of the HSF cytoplasm (B1-B3). ADORA2B is distributed in the cytoplasm and nucleus of HSF (C1-C3). ADORA3 is weakly expressed in the cytoplasm of HSF (D1-D3). Magnification: 400X.

### Tissue source

This study was approved by the Ethics Committee of Sun Yat-sen University in China and complied with the tenets of the Declaration of Helsinki for Research Involving Human Tissue. Four healthy, normal, adult human eyes from donors ranging 20–25 years of age were obtained from the eye bank of Zhongshan Ophthalmic Center (Sun Yat-sen University) and were numbered 1, 2, 3, and 4.

### Frozen section of human sclera preparation

From donor eyeball number 1 and 2, the anterior segment, the choroid, and the retina were removed. The posterior sclera was cut into 5×5 mm^2^ pieces, embedded with optimum cutting temperature compound (OCT), and cut into 5 µm sections at −20 °C. They were then tiled onto carrier slices, fixed with cool acetone for 15 min, air-dried, and kept frozen at −20 °C until use.

**Figure 3:**
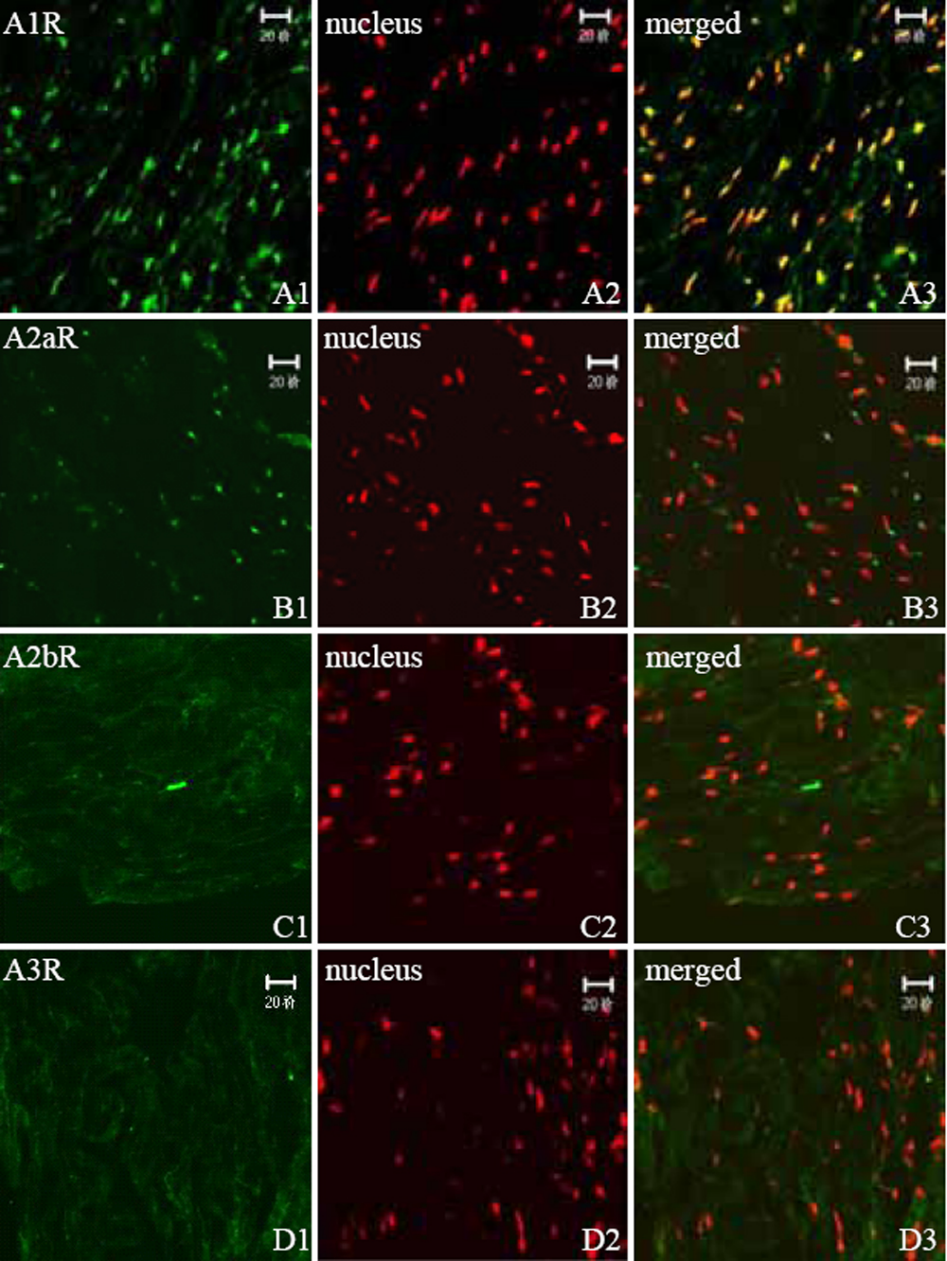
Distribution of ADORA1, ADORA2A, ADORA2B, and ADORA3 in human sclera using indirect immunofluorescence. FITC-marked the secondary antibody (green; 1) and PI dyed the nucleus (red; 2). (1) and (2) are combined into (3). ADORA1 is strongly condensed in the nucleus of HSF (A1-A3). ADORA2A is mainly distributed on one side of the HSF cytoplasm (B1-B3). ADORA2B and ADORA3 are distributed in the cytoplasm of HSF (C1-C3 and D1-D3).

### Human scleral fibroblast isolation, culture, and identification

Donor eyeball number 3 and 4 were washed immediately in HBSS containing penicillin (200 µg/ml) and gentamicin sulfate (400 µg/ml). The retinas and choroids were removed from the sclera. The sclera was trimmed into pieces approximately 1×1 mm in size, placed in 75 mm^2^ plastic culture bottles in DMEM with 1X antibiotic/antimycotic and 10% FBS, and incubated at 37 °C in a humidified incubator containing 5% CO_2_. The growth medium was changed twice a week. When a heavy primary monolayer was achieved, cells were trypsinized in 0.25% trypsin/EDTA solution in PBS at room temperature for 2 min and subcultured at a split ratio of 1:3 in a 75 mm^2^ plastic bottle. The third passage of fibroblasts was used for this experiment. The fibroblasts were grown on cover glasses in six well plates until 70%–80% confluence occurred. The cells were washed with PBS three times, fixed with cool acetone for 15 min, air-dried, and kept frozen at −20 °C until use.

The purity of fibroblast cell cultures was confirmed by staining for vimentin and stain resistance for cytokeratin, desmin, and S-100 in the indirect immunofluorescence procedure described below. The morphology of HSF was observed with light microscopy.

### Indirect immunofluorescence

The slices were washed three times with PBS, covered with 10% normal goat serum diluted in PBS, and incubated for 20 min at 37 °C. The slices were then incubated at 4 °C overnight with the primary antibodies (anti-vimentin, anti-cytokeratin, anti-desmin, anti-S-100, anti-ADORA1, anti-ADORA2A, anti-ADORA2B, and anti-ADORA3) diluted at 1:500 in PBS. A negative control was run in which the cells were incubated in PBS without the primary antibodies. The slices of both the antibody-treated and the negative control samples were washed with PBS and exposed to fluorescein isothiocyanate-conjugated (FITC) goat anti-rabbit IgG antibodies diluted at 1:50 in PBS at 37 °C for 30 min. The slices were again washed with PBS three times and added to propidium iodide (PI) for 5 min to stain the cell nuclei red. Immunofluorescent images were taken using a confocal microscope (LSM 510 META, Carl Zeiss, Jena, Germany).

### Western blot analysis

HSF were cultured in DMEM/F12 for 48 h and harvested; human sclera were shivered into pieces and centrifuged (15,000x g) at 4 °C for 1 h. HSF lysates were prepared in a lysis buffer and boiled in a sample buffer for 5 min. The protein concentration was detected by BAC kits. Protein (40 μg) was loaded in each lane of 10% SDS-polyacrylamide gels, transferred onto polyvinylidene difluoride membranes for electrophoresis, blocked in TBST (5% fat-free dry milk, 0.1% Tween 20, 150mM NaCl, and 50 mM Tris at pH 7.5) for 1 h. The membranes were exposed to 1 µg/ml of anti-ADORA1, anti-ADORA2A, anti-ADORA2B, and anti-ADORA3 polyclonal antibody and incubated overnight at 4 °C. They were then incubated with a secondary horseradish peroxidase-labeled antibody for 1 h. Protein bands were visualized with the use of a chemiluminescence Phototope(R)-HRP Western Blot detection system (Cell Signaling Technology, Inc., Danvers, MA) and exposed onto a negative film, developed, and fixed. The film was scanned and analyzed with Gel-Pro Analyzer software.

### Statistical analysis

Statistical analysis was performed using Student's two-tailed *t*-test and by analysis of variance (ANOVA) followed by the Sheffe F-test.

## Results

### Identification of human scleral fibroblasts

As shown in Figure 1, the cytoplasm of the studied cells strongly expressed vimentin protein but did not express keratin, desmin, or S-100, and the cells were thereby identified as fibroblasts. The HSF migrated from the pieces of sclera tissue and populated the surroundings. The cells exhibited a uniform, fusiform shape. Cell growth was compact and arranged in a vortex or radiating pattern.

### Expression of adenosine receptors in human scleral fibroblast

As shown in Figure 2, ADORA1 was only expressed and distributed in the nucleus of HSF. ADORA2A was expressed mainly on one side of the cytoplasm of HSF. ADORA2B was uniformly expressed in the cytoplasm and nucleus of HSF. ADORA3 was slightly expressed nn the cytoplasm of HSF.

### Expression of adenosine receptors in human sclera

As shown in Figure 3, ADORA1 was expressed mainly in the nucleus of HSF and slightly expressed in the cytoplasm of HSF. ADORA2A was expressed mainly on one side of the cytoplasm of HSF. ADORA2B and ADORA3 were uniformly expressed in the extracellular matrix and the cytoplasm of HSF.

### Western blot results

**Figure 4:**
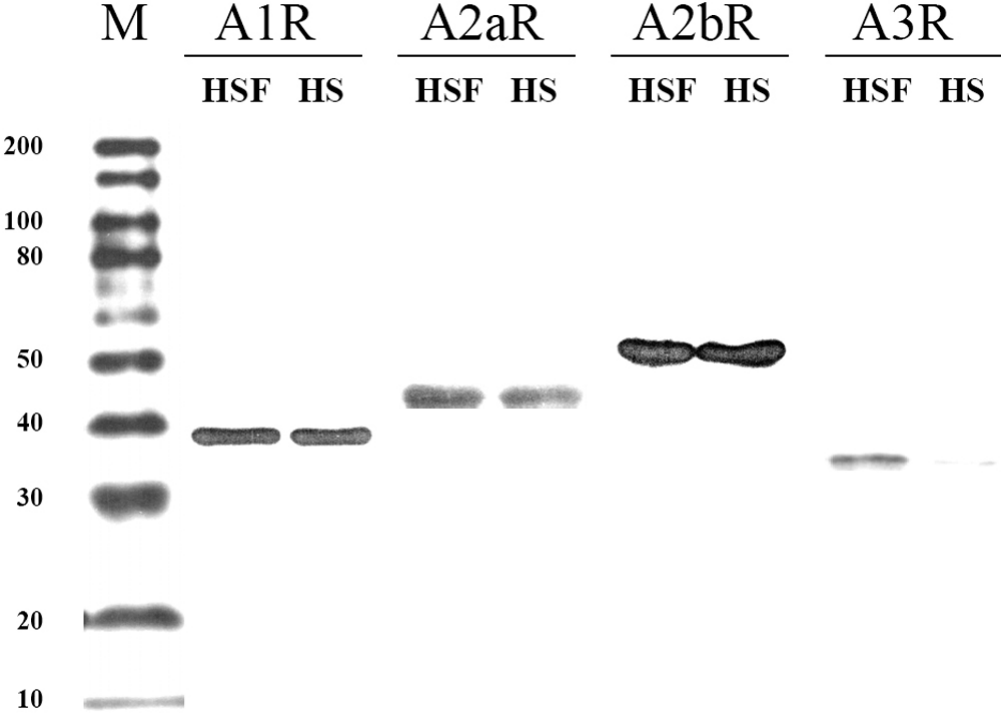
Four subtypes of ADOR expressed in HSF and human sclera (HS). The marker ladder is from 10 kDa to 200 kDa. The molecular weight of ADORA1 is about 39 kDa, ADORA2A is 45 kDa, ADORA2B is about 52 kDa, and ADORA3 is nearly 36 kDa. ADORA1 and ADORA2B are strongly expressed in both the HSF and human sclera, but ADORA2A and ADORA3 are only weakly expressed in the human sclera.

As shown in Figure 4, four subtypes of ADOR protein were found in HSF and human sclera. The approximate molecular weight for ADORA1, ADORA2A, ADORA2B, and ADORA3 was 39 kDa, 45 kDa, 52 kDa, and 36 kDa, respectively. ADORA1 and ADORA2B were strongly expressed in both the HSF and human sclera. ADORA2A and ADORA3 were also expressed in both the HSF and human sclera but only weakly in the latter. In particular, ADORA3 was very weakly expressed in the human sclera.

## Discussion

The identity of the cultured HSF was confirmed by positive indirect immunofluorescence reactivity to vimentin and by lack of reactivity to keratin, S-100 protein, and desmin [11-14].

The expression of ADORs in HSF has not been previously reported. Our finding of only a weak expression of ADORA3 in HSF is in accordance with earlier findings of no ADORA3 expression in rat eyes [9]. The varying localization of the ADORs in HSF, such as ADORA1 in the nucleus, ADORA2A in one side of the cytoplasm, ADORA2B weakly expressed in both cytoplasm and nuclei, and ADORA3 weakly expressed in the cytoplasm, can be ascribed to different cellular functions of these receptor subtypes. Different subcellular localization of adenosine receptors has been reported previously. In both dendrites and axons of rat striatal neurons, ultrastructural analysis has identified the adenosine ADORA2A receptor localized in the plasma membrane, throughout the cytoplasm, and around intracellular membranous structures [15].

Adenosine, a product of ATP catabolism, is a potent regulator of a multitude of cellular and organ functions via interaction with ADORs present in every type of tissue. ADORs are coupled to mitogen-activated protein kinases and may therefore play a role in cell cycle progression, proliferation, and differentiation [16].

Adenosine plays an important role as a neuromodulator in mammals but also affects supportive cells in the central nervous system. Thus, adenosine induces rapid glycogenolysis followed by increased glycogen synthesis in primary astrocytes. The effect seems to be mediated by ADORA2B adenosine receptors. In response to traumatic and chemical brain injury involving the upregulation of enzymes, cytoskeletal proteins, transforming growth factor β (TGFβ), nerve growth factor, basic growth factor, and growth factor receptors, reactive astrogliosis is mediated by ADORA2A and possibly ADORA3 whereas ADORA1 inhibits astrocyte proliferation [17]. ADORA2A antagonists prevent astrogliosis induced in vitro by basic fibroblast growth factors [18].

ADORA2A agonists enhance the rate of matrix formation in wound healing. Human dermal fibroblasts express ADORA1, ADORA2A, and ADORA2B [19]. ADORA2B on synovial fibroblasts diminish the release of matrix metalloproteases, and on pulmonary fibroblasts, they increase collagen production and diminish metalloprotease production. Similarly, in human skin and liver cells, ADORA2A promotes collagen synthesis and diminishes metalloprotease secretion [20]. ADORA2B, however, diminishes collagen production and cell proliferation of cardiac fibroblasts [21,22]. Overstimulation of ADORA2A is probably a pathogenetic factor in scleroderma and hepatic fibrosis [19]. The cytokine, TGF-β, upregulates adenosine ADORA2A expression in human dermal fibroblasts [19]. Interestingly, downregulation of TGF-β is observed in mammals during the progression of myopia induced by form deprivation [23].

Topically applied muscarine acetylcholine antagonists have been shown to reduce myopia progression and axial eye growth [24-26]. The mechanism of action of atropine and pirenzepine has not been established, but M1 to M5 muscarinic acetylcholine receptors have been identified in HSF, and HSF are therefore a potential site of action of these drugs [27-29]. M2, M4, and M5 muscarinics have also been identified in human skin fibroblasts [30]. Interestingly, in many cell systems, muscarinics and ADORs interact. Thus, M2 muscarinic receptor activation inhibits ADOR-evoked relaxation of the rat detrusor muscle, and M1 muscarinic and ADORA2A receptors are mutually exclusive at facilitating acetylcholine release from rat motor endplate motoneurons [31,32]. In the central nervous system, ADORA2A agonists increase muscarinic acetylcholine receptor-mediated neurotransmission [33,34], and low doses of the non-selective ADOR antagonist caffeine enhances the anticataleptic actions of muscarinic antagonists in rats [35]. It is therefore conceivable that adenosine and muscarinic acetylcholine receptors on scleral fibroblasts also interact and that blocking of ADORs induces extracellular matrix composition changes due to modulation of muscarinic acetylcholine transmission or vice versa. It is likewise conceivable that simultaneous blocking of both adenosine and muscarinic acetylcholine receptors has an additive effect on the production of extracellular matrix by the scleral fibroblast.

The finding of ADOR expression in HSF signified that adenosine and ADOR may play a role in scleral remodeling during eye growth. Further research on the role of different ADOR subtypes is needed.
